# A Comparative Study of Condylar Bone Pathology in Patients with and without Temporomandibular Joint Disorders Using Orthopantomography

**DOI:** 10.3390/jcm12185802

**Published:** 2023-09-06

**Authors:** Mohamed Jaber, Alaa Khalid, Amena Gamal, Raghad Faisal, Asok Mathew, Mohamed Ingafou

**Affiliations:** 1 Department of Clinical Sciences, College of Dentistry, Ajman University, Ajman P.O. Box 346, United Arab Emirates; mohamed.jaber@ajman.ac.ae (M.J.); m.ingagou@ajman.ac.ae (M.I.); 2 Centre of Medical and Bio-Allied Health Sciences Research (CMBHSR), Ajman University, Ajman P.O. Box 346, United Arab Emirates

**Keywords:** temporomandibular disorder, temporomandibular joint, bruxism, condylar head, erosion

## Abstract

This study aimed to compare condylar bony pathology in patients with and without temporomandibular joint disorder (TMD) using orthopantomography at Ajman University dental clinics between 2017 and 2021. Patient data from the Ajman University archives were collected after obtaining ethical approval. OPG (orthopantomogram) views were evaluated for potential TMJ pathology. Three independent observers underwent calibration and image analysis, with their agreement level calculated using Kappa statistics (score 0.781). Condylar changes were coded from 0 to 6. Statistical tests such as the Mann–Whitney Test, Kruskal–Wallis test, Spearman’s correlation, and logistic regression analysis were used to analyze the data. The inter-examiner reliability for OPG was 0.903, and intra-examiner reliability was 0.908. The most common condylar bony changes observed in OPG views were flattening and osteophyte. Female participants had a higher prevalence of all bony changes. Temporomandibular Disorder (TMD) can manifest with symptomatic and detectable bony changes in OPG views. The prevalence of temporomandibular disorder appeared similar between genders, but differences were observed regarding the number of teeth lost, with unilateral tooth loss being more common. Interestingly, bruxism did not seem to significantly impact of temporomandibular disorder patients.

## 1. Introduction

Temporomandibular joint disorders (TMD) are complex health conditions that result from alterations in the structure or function of the masticatory muscles, temporomandibular joint (TMJ), and associated structures (Buescher, 2007). of temporomandibular disorder encompasses a range of conditions characterized by TMJ or surrounding tissue pain, mandibular functional limitations, or clicking during TMJ movement [1]

The term “temporomandibular joint (TMJ) syndrome” was initially defined by Costen in 1936, referring to a muscular–skeletal pain disease that causes pain projected to various head and neck regions due to hypertonia of the masticatory muscles [2]. In the mid-1950s, theories surrounding TMJ pathology focused on the relationship between temporomandibular disorder and occlusion [3].

Laskin proposed that muscle spasms and fatigue caused by chronic oral habits contribute to the symptoms of mandibular pain dysfunction, highlighting the multifactorial nature of TMJ disorders [3]. Recent advances in neurophysiology have introduced the concept of central nervous system (CNS) plasticity and its role in persistent pain, emphasizing the importance of pain management from a psychosocial and behavioral perspective [3].

The etiology of TMJ disorders remains uncertain, but it is likely multifactorial. Predisposing, initiating, and perpetuating factors contribute to the development of TMD [4]. Predisposing factors include systemic, psychological, structural, and genetic factors that increase the risk of TMD. Initiating factors trigger the onset of the disorder, such as trauma or overloading of the joint structures due to parafunctional habits. Perpetuating factors interfere with healing or complicate management, including mechanical and muscular stress [4]. Abnormal occlusion, parafunctional habits, stress, anxiety, and intra-articular disk abnormalities can result in capsule inflammation or damage and muscle pain or spasms [2]. However, the role of occlusion in TMJ symptoms has been questioned, as abnormal dental occlusion is equally common in individuals with and without TMJ symptoms, and occlusal correction does not reliably improve symptoms or signs of TMJ disorders [2].

Anxiety, stress, and emotional disturbances may aggravate TMJ disorders, particularly in patients with chronic pain [5].

The prevalence of temporomandibular disorder is estimated to be greater than 5% of the population, with a peak occurrence between 20 and 40 years old. It is more common in women than in men [6]. Prevalence rates vary across studies, with approximately 31% of adults and elderly individuals and 11% of children and adolescents experiencing TMD.

The prevalence and treatment of TMD can vary significantly due to cultural, geographical, and socioeconomic factors. These factors can influence the understanding, prevalence, and management of TMD in different populations. By identifying these limitations, healthcare professionals can develop more effective strategies for diagnosing and treating TMD across diverse populations.

In some cultures, TMD symptoms may be attributed to spiritual or superstitious beliefs, leading to delayed or inappropriate treatment-seeking behavior [7]. The stigma surrounding mental health issues might also discourage individuals from seeking help for TMD-related psychological factors, such as stress and anxiety. Cultural dietary habits, such as chewy or hard foods, may exert excessive pressure on the TMJ, contributing to TMD prevalence in specific populations [8]. Additionally, cultural practices like habitual teeth clenching or grinding may exacerbate TMD symptoms. Geographical variations in climate and environmental factors may influence TMD prevalence. Extreme cold or damp climates could potentially exacerbate TMD symptoms, especially in individuals with pre-existing joint issues [9]. Access to Healthcare: Rural or remote populations may face challenges in accessing healthcare facilities and specialists skilled in diagnosing and treating TMD [10]. This limited access can lead to underdiagnosed and undertreated TMD cases. Socioeconomic disparities may impact an individual’s ability to seek timely and appropriate treatment for TMD. Costs associated with diagnosis, imaging, and specialist consultations may be prohibitive for individuals from lower-income backgrounds [11].

The Diagnostic Criteria for Temporomandibular Disorders (DC/TMD) Axis I classification system provides a comprehensive overview of the different types of temporomandibular disorders (TMD). The classification, as recommended by Schiffman et al. (2014) [12], is widely used for both clinical and research purposes. The DC/TMD Axis I classification system provides a standardized and systematic approach to diagnosing and classifying different TMD conditions. It aids clinicians in accurately identifying the specific TMD subgroup a patient belongs to, leading to more targeted and effective treatment strategies.

According to the DC/TMD Axis I, TMD can be divided into the following three groups:

- Group I: Muscle Disorders Group I includes muscle disorders, which involve abnormalities or dysfunctions of the muscles of mastication and the surrounding structures. This group encompasses myofascial pain, both with and without mouth-opening limitation. Myofascial pain is characterized by pain and tenderness in the muscles of the jaw, head, and neck region, and it may be associated with trigger points and limited mouth opening due to muscle tightness or spasms.
- Group II: Disc Displacement with or without Reduction and Mouth Opening Limitation Group II consists of disc displacement disorders, which involve abnormal movements or positions of the articular disc within the temporomandibular joint (TMJ). This group is further divided into subgroups based on whether the disc displacement is accompanied by joint reduction (return to normal position upon mouth opening) or without reduction (no return to normal position). Patients in this group may experience limitations in mouth opening due to the disc’s position interfering with joint movement.
- Group III: Arthralgia, Arthritis, and Arthrosis Group III comprises arthralgia, arthritis, and arthrosis. Arthralgia refers to joint pain without evidence of structural abnormalities, while arthritis involves inflammation of the TMJ. Arthrosis, on the other hand, refers to degenerative changes in the joint, such as osteoarthritis. Patients in this group may experience joint pain, swelling, and functional limitations due to joint pathology.

Valesan et al. (2021) [13] conducted a comprehensive systematic review and meta-analysis to determine the global prevalence of TMD. The study analyzed data from various population-based studies published up to 2021. The results indicated that TMD is a highly prevalent condition, affecting a substantial number of individuals worldwide. The overall pooled prevalence of TMD was estimated to be approximately 14.8%. This prevalence varied across different geographic regions, with some areas reporting higher rates of TMD than others. Jin et al. (2016) [14] conducted a systematic review to assess the global burden of musculoskeletal disorders, including TMD. The study highlighted that TMD is the second most common musculoskeletal disorder causing pain and disability, after low back pain. This finding underscores the significant impact of TMD on individuals’ quality of life and its relevance as a public health concern.

The epidemiological data from Valesan et al. (2021) [13] and Jin et al. (2016) [14] provide important insights into the prevalence and burden of TMD. The high prevalence rate of TMD reported by Valesan et al. (2021) [13] emphasizes the need for effective prevention, early diagnosis, and appropriate management strategies to address this widespread health issue. Additionally, Jin et al. (2016) [14] highlighted the significant impact of TMD on pain and disability, underscoring the importance of early intervention and comprehensive care to improve patients’ well-being.

Central sensitization is a neuroplastic phenomenon characterized by an increased sensitivity of the central nervous system (CNS) neurons to nociceptive stimuli. In conditions like TMD, where chronic pain persists, the CNS undergoes changes that result in an amplification of pain signals and altered pain processing. This leads to expanded pain perception and increased responsiveness to pain, a condition known as allodynia [15,16].

It was reported that overlapping symptoms in TMD and Other Chronic Pain Conditions may happen, such as TMD patients experiencing headaches, especially tension-type headaches and migraines. The convergence of nociceptive inputs from the trigeminal nerve, cervical spine, and other cranial nerves onto the same neurons in the brainstem can lead to a cross-sensitization phenomenon, where pain signals from TMD can amplify headaches and vice versa. Fibromyalgia is a chronic pain disorder characterized by widespread musculoskeletal pain, fatigue, and tender points. TMD patients may exhibit similar tender points and widespread pain due to the shared mechanism of central sensitization. Moreover, both conditions involve dysregulation of pain processing pathways, leading to heightened pain responses. Certain neurological conditions, such as neuropathic pain syndromes and complex regional pain syndrome (CRPS), can present with symptoms similar to those seen in TMD. Central sensitization is a common underlying mechanism in these conditions, resulting in allodynia and hyperalgesia. Allodynia refers to the perception of pain from non-painful stimuli, such as light touch or pressure. TMD patients may experience allodynia in response to innocuous stimuli on the face or jaw, indicating the involvement of central sensitization. Hyperalgesia, on the other hand, is an increased sensitivity to painful stimuli. TMD patients may exhibit hyperalgesia in response to jaw movements or dental procedures, further supporting the central sensitization hypothesis.

### Conservative Treatment Modalities for TMD

Physical Therapy and Exercise: Physical therapy and exercise have emerged as important components of conservative TMD management. Studies, such as those by Pihut et al. (2021) [17] and Santos et al. (2022) [18], have demonstrated the benefits of targeted exercises, jaw stretching, and manual therapy in improving mouth opening, reducing pain, and enhancing jaw function. These approaches promote muscle relaxation, joint mobility, and overall musculoskeletal health.

Occlusal Splints: Occlusal splints, also known as bite guards or oral appliances, have been extensively studied for TMD management. Research by Al-Saleh et al. (2020) [19] and Li et al. (2021) suggests that properly fitted splints can help reduce parafunctional habits, minimize joint loading, and provide pain relief. Different types of splints, such as stabilization splints and anterior repositioning splints, have shown promising results in various TMD subtypes [20].

Pharmacological Interventions: Recent studies have investigated the role of medications in conservative TMD treatment. Topical analgesics, nonsteroidal anti-inflammatory drugs (NSAIDs), and muscle relaxants have been explored for their potential to alleviate pain and inflammation. The work of Devenney et al. (2020) and Sharma et al. (2022) indicates that pharmacological interventions can complement other conservative therapies, providing short-term relief and improving patients’ comfort [21,22].

Mind–body Approaches: Mind–body techniques, such as cognitive-behavioral therapy (CBT) and relaxation techniques, have gained attention in TMD management. Literature by Harper et al. (2021) [23] and Guglielmotti et al. (2022) [24] suggests that addressing psychological factors, stress, and anxiety can positively impact pain perception and help manage TMD symptoms.

Self-Management Strategies: Empowering patients with self-management strategies has become a focal point in conservative TMD treatment. Educational programs and digital health interventions, as shown in studies by Ma et al. (2020) and Choi et al. (2021), offer patients valuable tools to understand their condition, adopt healthy habits, and actively participate in their recovery [25,26].

Various radiographic techniques can be used to evaluate TMJ conditions. Orthopantomography provides an overall screening evaluation of the TMJs, showing the surrounding anatomy, such as the articulating surfaces, glenoid fossa, mandibular condyles, and other areas. Magnetic resonance imaging is effective for evaluating TMJ soft tissues and dynamic joint function without ionizing radiation. Nuclear medicine techniques, such as technetium-99 scanning, can be used to assess active bone metabolism in the TMJ, although interpretation can be challenging [27].

The TMJ is susceptible to various complications and disorders due to its complex nature and the involvement of different types of tissues. These disorders can be categorized as derangement disorders of the condyle-disc complex, structural incompatibility between different tissues, and inflammatory joint disorders [28,29]. Inflammatory joint disorders involve the inflammation of the synovial tissues and joint capsule, including conditions like synovitis, capsulitis, retro-discitis, and arthritis. Retro-discitis occurs when retro-discal tissues are encroached upon by the condyle due to altered disc position [30].

Less common conditions that affect the TMJ include condylar hyperplasia and hypoplasia, which are characterized by noticeably larger or smaller condyles, respectively. Condylar hypoplasia can result from various causes, such as trauma, infection, or exposure to radiation during development [31].

The objectives of this study are:

- To assess the prevalence of TMJ bony changes among patients with and without TMD.
- To focus on evaluating the diagnostic capability of orthopantomography (OPG) in detecting bone pathology associated with TMD.

In our study, the diagnosis of Temporomandibular Disorder (TMD) was primarily based on a comprehensive clinical examination and imaging with Orthopantomography (OPG). TMD is a complex condition with various signs and symptoms, making an accurate diagnosis crucial for appropriate treatment planning. The clinical examination allowed us to assess the patient’s history, evaluate their reported symptoms, and perform a thorough assessment of their temporomandibular joint (TMJ) and masticatory muscles [32,33].

The OPG was the primary imaging modality used for the diagnosis of TMD in our study. An OPG is a two-dimensional radiographic image that provides a panoramic view of the maxillofacial region, including the TMJ. It allows visualization of the condyles, articular eminences, and mandibular fossae, providing valuable information about joint morphology, condylar position, and potential structural abnormalities. OPG is a relatively simple and cost-effective imaging technique commonly used in dental practices to aid in the assessment of TMD cases [34,35,36].

While OPG is a useful initial screening tool for TMD diagnosis, we acknowledge that additional imaging exams, such as Magnetic Resonance Imaging (MRI) or Cone Beam Computed Tomography (CBCT), could provide more detailed and comprehensive information. MRI is particularly helpful in assessing soft tissues, such as the articular disc, ligaments, and surrounding muscles, which are not clearly visible on OPG. CBCT, on the other hand, offers three-dimensional images, providing a more accurate assessment of the TMJ’s bony structures and condylar position [37,38,39,40].

The decision to use OPG as the primary imaging modality was based on factors such as patient comfort, availability, and less radiation exposure [41].

## 2. Material and Method

The College Research Ethics Committee carries out the ethical reviews of all research projects conducted at Ajman University, and the study was channeled through the REC to gain approval. (Reference number: D-H-S-2021-OCT-9-4).

The data were collected from the patient archives of Ajman University. The files selected were from the years 2017–2021. Any file that was not within the mentioned year range was excluded from the research. The inclusion criteria of the case group were that the patient must have been previously diagnosed with temporomandibular disorder regardless of what type of TMJ disorder it is. A copy of the patient’s file was taken and stored confidentially. The file basically contained all the necessary information for this research, which includes the age, gender, history, chief complaint, medical history, dental history, family history, habits, extraoral examination, which includes the lymph nodes status, the TMJ examination findings, and a diagnostic dental chart. Additionally, it was important that each patient’s file included in the study had Orthopantomogram (OPG) taken prior for the evaluation. The OPG’s were collected and stored from the universities X ray program software Scanora. Any patient’s file that did not contain an OPG was excluded from the research. The diagnosis was based on the Diagnostic Criteria for TMD (DC/TMD) guidelines. The following parameters were used for this research such as age, gender, medical history, dental history, teeth missing, number of teeth missing, history of temporomandibular disorder, TMJ side affected, history of bruxism and TMJ pathologies were coded from 0 to 6, representing different pathologies (0: Normal, 1: Erosion, 2: Flattening, 3: Osteophyte, 4: Sclerosis, 5: Resorption, 6: Cyst, and 7: other changes). For the age, we divided the study group into four groups which is less than 50 years old, 50–60 years, 60–70 years, greater than 70 years old. Regarding the teeth missing, it was judged based on if the teeth are missing unilaterally, bilaterally, or no missing teeth. Number of missing teeth was classified into three groups which are 2–3 missing teeth, more than 3 missing teeth or no missing teeth. The history of temporomandibular disorder was a very important criteria as it was used to help us to differentiate between the case and control group. The controls were indeed selected from radiographic studies of patients attending Ajman University dental clinics for reasons other than TMD. We acknowledge the potential selection bias this may introduce.

In pathology, image evaluation included several conditions such as resorption of the condylar head, flattening, osteophytes, hyperplasia, agenesis, cyst, and calcification. The sample size (*n*) was calculated via the online Open Epi link using the Kish formula for sample size estimation, choosing a 95% significance level, a 5% margin of error, and a 50% response rate; the representative total sample size is 383.

The sample size finally included a total of 210 patients attending Ajman University dental clinic, and the study group was divided into two groups: 105 subjects in the case group and 105 in the control group. The discrepancy between the recruited sample and the ideal sample size calculated can cause inconsistency in the results.

For proper evaluation of the collected OPG views, two assessors were initially trained using a maxillofacial radiologist to calibrate. Inter- and intra-observer agreements were calculated using the Kappa value and were found to be satisfactory (0.781).

### Statistical Analysis

The data collected were statistically analyzed with the SPSS version.28 (SPSS, Inc., Chicago, IL, USA). Collected data were organized and tabulated as descriptive results in terms of frequency and percentages and then included the gender, age, medical and dental history, and pathology for the case and control groups, respectively. Chi-square test (X^2^) was applied to know the association between study variables and different study characters.

Mann–Whitney U Test and Kruskal–Wallis test were used to determine significant difference of the distribution of all study parameters across study categories. Spearman’s correlation was used to compares the strength of the effect of each independent variable to the dependent one. Equally, multilinear logistic regression analysis used to identify factors associated with case and control groups. The statistical significance (*p*-value) was set in this study at below 0.05 with 95% confidence interval

## 3. Results

The demographic distribution of the data shown in Table 1 revealed that the age variable in the case group (105 cases) had a frequency of 85 patients (81%) in patients aged less than 50 years and a frequency of 2 patients (1.9%) for patients aged above 70 years. On the other hand, the age variable in the control group reported a frequency of 85 patients (81%) for patients aged less than 50 years and one patient (1%) for patients aged above 70 years (Table 1).

The second variable studied is gender; it was reported that the patients from the case group had a frequency of 58 male patients (55.2%) compared to 47 female patients (44.8). In the control group, male gender had a frequency of 62 (59%) patients and 43 (41%) female patients.

The third variable studied was the medical history. In the case group, it was reported that 80 out of the 105 cases (76.2%), while 91 out of 105 controls (86.7%) were medically fit.

Dental history is the fourth variable that was included in this study, in which 81 (77.1%) of them were regular visitors in the dental facility for the case group and 80 (76.2%) for the control group.

One of the criteria that was included in this study was the incidence of missing teeth. In these cases, the patients with a unilateral loss of teeth had a frequency of 43 patients (41%), while the frequency for patients with no tooth loss was 25 patients (23.8%). On the opposite side, the controls had a frequency of 57 patients (54.3%) for patients with no tooth loss and 22 patients (21%) for patients with unilateral loss of teeth (Table 2).

The number of teeth missing between the cases and controls was included in this case–control study. Here, you can see that in the cases, patients with 2–3 missing teeth had a frequency of 44 patients (41.9%) while the patients with no tooth loss were 25 patients (23.8%). In the controls, the patients with no tooth loss were 57 patients (54.3%), while patients with 2–3 missing teeth were 21 patients (20%).

Moreover, regarding TMJ affected, the Case group had both TMJs affected in 45 patients (42.9%), followed by the right TMJ in 31 patients (29.5%), then the left TMJ in 29 patients (27.6%).

The frequency of pathologies among our sample is presented in Table 3. 32 (30.5%) individuals out of 105 presented with condylar flattening. At the same time, the least recorded pathology (1%) was the presence of cysts in TMJ. Additionally, all of our control group presented with normal and healthy condyles (Table 3).

Likewise, bruxism also showed variable prevalence. According to our calculations, 18 out of 105 (17%) individuals within our case group experienced bruxism (Table 4).

Concerning medical history, in most of both case and control groups, 86.7% of the control and 76.2 of the case group did not present with any systematic illnesses. The most reoccurring illness within the control group was diabetes where 10 individuals had it. On the other hand, hypertension was the prevalent disease in the case group with 6 individuals presenting with it (Table 5).

One of the most critical variables observed in the present was the presence of missing teeth and the symptoms associated with TMJ. When looking at the presence of missing teeth in our case group with temporomandibular disorder, 80 subjects had missing teeth. This is very dissimilar to the control group, where 57 individuals did not present with missing teeth. As seen from Table 5 the maximum number of teeth missing is observed to be unilaterally present both in case and control group but with a very significant statistical difference of <0.001.

When looking at the number of teeth missing, we noticed that closer to one-half of our case group had 2–3 missing teeth (41.9%), with the lesser majority having more than 3 missing teeth and only 25 subjects having a full complement of teeth. In contrast, more than half of our control group were fully dentate (54.3%), and a similar distribution of 20% and 25% for 2–3 or >3 missing teeth, respectively, with a significant difference between the two groups (*p* < 0.001) (Table 5).

Regarding the pattern of TMJ affected, more than one-third of our case group presented with pathology in both the condyles (42.9%), with the remaining individuals sharing a similar 29.5% and 27.6% for the right and left condyles, respectively, therefore creating a statistically significant *p* value of <0.001 between case and control (Table 6).

When referring to the exact pathology detected, we can see that 32 out of 105 patients (almost 1/3rd of the cases) had condylar flattening (30.5%), followed by 20% of condylar resorption.

Less than one-fifth presented with no pathology in the condyles (19%), proceeding the 13.3% with condylar hyperplasia observed according to the recognizable and distinct asymmetrical enlargement of the mandibular condyle that is undoubtedly a characteristic feature of this pathology. Collectively, the rarer pathologies spotted were bifid condyles, osteophytes and cysts with percentages of 9.5%, 6.7%, and only 1%, respectively (Table 7). The OPG findings of bifid condyles displayed grooves or depressions surrounding the mid-line of the condylar heads, thus create a double appearance. Osteophytes, on the other hand, were detected based on the findings of marginal hypertrophy along with exophytic angular radio-opaque bony formations emerging from the condylar heads of these patients. Cysts were confirmed by their manifestation of solitary, well circumscribed radiolucency surrounded with opaque borders.

Interestingly, nearly all of our control group did not have bruxism with a bulk mass of 104 out of 105 patients 99%. On the other hand, only 87 patients in our case group did not present with such condition (82.9%) that is to say more than two thirds of people in our study presenting with temporomandibular disorder seem to have bruxism. According to our test results there’s a significant statistical difference between the two groups when compared with history of bruxism with a value of (*p* < 0.001) (Table 8).

There is a very high positive correlation between the case group and the TMJ affected, with a correlation value of 0.934 and a highly significant level of *p* < 0.001. A moderate correlation of a value of 0.605 can be seen between the case group and the pathology category, with a highly significant level of *p* < 0.001. A low correlation can be seen between the case group and the teeth missing and the number of teeth missing. There is a high significant level between them of a value *p* < 0.001. Regarding age, gender, and medical and dental history, there is a very weak correlation between them and between the number of missing teeth, TMJ-affected pathology, and bruxism. There is no significance between them as most of the values are *p* > 0.05 (Table 9).

A very high correlation can be seen between the number of teeth missing, with a correlation value of 0.916. The significance value between the teeth missing and the number of teeth missing is *p* < 0.001, which is statistically significant.

Regarding the correlation between the teeth missing and TMJ-affected pathology and bruxism, we can clearly see that the correlation is neglectable. The significance level between the teeth missing and the TMJ affected is high, being *p* < 0.001. There is a significant between teeth missing and the pathology of a value of *p* = 0.023. However, there is no significant statistical difference between the teeth missing and bruxism. There is a low correlation between the number of missing teeth and the TMJ affected with a value of 0.309. However, there’s a high significance value between the two categories, *p* < 0.001 (Table 9).

To add, we can see that there is a neglectable correlation between the number of missing teeth and the pathology and bruxism category of values of *p* = 0.133 and *p* = 0.011, respectively. A significant value exists between number of teeth missing and pathology. However, there is no significance between number of teeth missing and bruxism. Regarding the correlation between the TMJ affected and the pathology, a moderate correlation can be seen between those categories of a value of 0.557 with a highly significant level of *p* < 0.001. There is a neglectable correlation between the TMJ affected and bruxism of a value of 0.557 with a highly significant level of *p* < 0.001. The correlation between the pathology category and the bruxism is low since the value is 0.310 although the significant level is said to be high since its *p* < 0.001 (Table 9).

As mentioned in the table above, we can assume that 18 out of the 105 case groups are at risk of developing bruxism in the future compared to the control group. However, 87 out of the case group will not develop bruxism. In the control group, most of the patients will not be at risk of developing bruxism in the future. The estimated risk for the case and control group is a value of 21.517 (Table 10).

The case group has a 2.159 higher risk of having the right TMJ affected compared to the control group. Regarding gender, males have a higher risk 1.433 of having both their condyles affected compared to females. The case group is 0.300 times more at risk of having bilateral tooth loss compared to the control group, with a highly significant level of 0.001. Males tend to have a higher risk of bilateral tooth loss of 1.635 chance compared to females. However, there is no significant difference. The case group has a higher chance of developing hyperplasia with a ratio of 1.299 followed by flattening of a rate of 1.019. Both groups have no significant rate. Regarding gender, males tend to be at risk of developing hyperplasia rather than flattening compared to females (Table 11).

## 4. Discussion

In our study, 44% of temporomandibular disorder cases were females, and radiographic evidence of condylar resorption was found in 20% of the cases [42,43,44]. Likewise, Larry M. Wolford [42] reported that condylar resorption in the TMJ is more common in females than males, particularly in teenage girls during the pubertal growth phase. This may be due to hormonal changes and the presence of estrogen receptors in the TMJs of females [45,46].

Flattening of the TMJ condyle refers to a degenerative alteration caused by TMJ overload and the involvement of masseter and temporal muscles [47,48,49,50,51]. It manifests as the loss of the rounded contour surface of the condylar head or the articular eminence, primarily due to the destruction of fibrocartilage and bone.

In a study conducted at Pusan National University in Korea, examining condylar bony changes in patients with TMD, flattening was observed in 112 joints (25.5%) out of 220 patients, representing the third highest percentage among their findings [47]. In our study, flattening of the TMJ exhibited the highest percentage among all other pathologies. Radiographic evidence of condylar flattening was found in 32 patients (30.5%) out of the 105-case group. However, the prevalence of condylar bony changes in this study was lower than that reported in a study by Cevidanes et al., where condylar flattening was observed in 60% of the condyles, specifically 35 out of 58 condyles [48].

Osteophytes (bone spurs) in the TMJ were found to be rare; in this study, study 7 out of 105 individuals presented with this pathology (6.7%), which is in agreement with a prevalence of the condition in a Brazilian study [49].

Condylar hyperplasia, characterized by excessive bone growth, was reported to be more prevalent in women [50], but our study did not find a significant gender difference in its occurrence, the reason that the prevalence of condylar hyperplasia is twice in females compared to males might be explained by the influences of both estrogen and prolactin hormones, and these could act as an immunological stimulus promoting bony changes.

Synovial cysts of the condyle, which have similarities in appearance to ganglion cysts, were identified in 1% of cases in our study, primarily in females [51]. Another study reported a slightly higher prevalence in females with an average age of 46 years. The symptoms varied greatly between patients diagnosed with synovial cysts, but most of them have reported a history of trauma prior to its development.

Bifid mandibular condyle, a rare condition characterized by division or duplication of the condylar head, was found in 9.5% of cases in our study, whereas the prevalence was 0.3% in a Turkish study [52,53]. It was reported that the condition is usually unilateral and asymptomatic. The exact cause of bifid condyle development is still unknown. Some suggest that the blood supply to this region was impeded during development, while others are of the opinion that during the early period of development, condylar cartilage has undergone division by a fibrous septum. Additionally, infections, endocrine disturbances, radiation, and nutritional deficiencies were reported as some of the potential causes.

Regarding the pattern of TMJ affected, more than one-third of our case group presented with bilateral involvement (42.9%), with the remaining individuals sharing a similar 29.5% and 27.6% for the right and left condyles, respectively. 82.9% of the subjects, which is about two-thirds of the subjects in our study, presented with temporomandibular disorder without any history of bruxism. Ohlmann B et al. has studied the correlation of sleep bruxism and temporomandibular disorder the findings reveal that there is a significant correlation between sleep bruxism and tempromandibular disorders which was not observed in our study. [54]

Another interesting statistical finding from the study is that males have a higher risk (1.433 times) of having both their condyles affected compared to females and have a higher risk of bilateral tooth loss (1.635 times) compared to females. Malheiros AS et al. have studied the incidence of temporomandibular disorder edentulous patients and found that the degree of tooth loss is directly related to the signs and symptoms of TMD [55]. The above study agrees with our findings.

Orthopantomography (OPG) is a commonly used imaging technique in dentistry for diagnosing various dental conditions, including temporomandibular joint disorders (TMD). While OPG offers several advantages, it also has inherent limitations that can impact its accuracy and efficacy in the diagnosis of TMD [56,57]. By understanding these limitations, dental professionals can make informed decisions about the appropriate use of OPG in the context of TMD assessment.

TMD diagnosis is essential for determining appropriate treatment plans and improving patient outcomes. Orthopantomography (OPG) is a widely used imaging technique in dental practice, but it has certain limitations when applied to TMD diagnosis, which include limited Visualization of Soft Tissues. As a result, it provides limited information about the soft tissues, including the articular disc, ligaments, and muscles of the TMJ. This deficiency can lead to an incomplete assessment of TMD, as soft tissue abnormalities may be missed [12,58], and there is a lack of dynamic information. OPG is a static imaging modality and cannot assess the dynamic movements of the TMJ during mouth opening and closing. TMD often involves dysfunctional movements and clicking sounds, which are not evident in static OPG images. Dynamic imaging techniques like cone-beam computed tomography (CBCT) or magnetic resonance imaging (MRI) are more suitable for capturing such dynamic changes [58,59], limited evaluation of condylar position: OPG provides a two-dimensional representation of the TMJ, making it challenging to accurately evaluate the condylar position in all three dimensions. Condylar displacement, which is common in TMD, may be overlooked or inaccurately assessed with OPG alone [60], inadequate assessment of joint degeneration: OPG has limited sensitivity in detecting early stages of joint degeneration, which can be a significant factor in TMD. More advanced imaging techniques like CBCT or MRI are better equipped to identify subtle changes in joint structures associated with degeneration [57,60], and finally, OPG superimposes anatomical structures, making it difficult to differentiate between overlapping structures, especially in complex cases of TMD [57]. This may result in misinterpretation and misdiagnosis [61,62]. When any future studies are planned, it is always better to combine OPG data with more advanced imaging modalities, such as MRI, which will pave the way for more comprehensive analysis.

The limitation of this study is the small sample size of subjects above 50 years, which is due to the limited number of patients registered in the year 2020 because of the lockdown during COVID-19. In addition, the flow of patients afterward was significantly reduced due to the pandemic restrictions. The authors acknowledge the fact that this small sample size studied will not represent the entire population. We recommend expanding the age range to include patients aged 40 years and above, that would provide a more comprehensive analysis of the relationship between age and pathology in the TMJ. The authors feel that this limitation can be highlighted as a topic for future investigation.

## 5. Conclusions

Temporomandibular Disorder (TMD) is symptomatic in some patients, which may also have bony changes that can be detected using OPG views. The prevalence of temporomandibular disorders appeared to be similar between genders. However, there were differences in relation to number of teeth lost, with the majority of temporomandibular disorder patients experiencing unilateral tooth loss. Both condyles were commonly affected, with condylar flattening being the most prevalent pathology, followed by condylar resorption. Interestingly, bruxism did not appear to have a significant impact on temporomandibular disorder patients.

## Figures and Tables

**Table 1:** The frequency among the case and control in relation to gender, age, and medical/dental history.

		Case		Control	
		Frequency	Percent	Frequency	Percent
Age	<50 years old	85	81.0	85	81.0
	50–60 years old	13	12.4	16	15.2
	60–70 years old	5	4.8	3	2.9
	>70 years old	2	1.9	1	1.0
Gender	Male	58	55.2	62	59.0
	Female	47	44.8	43	41.0
Medical History	No medical history	80	76.2	91	86.7
	Hypertension	5	4.8	6	5.7
	Diabetes	10	9.5	4	3.8
	Bone pathology	2	1.9	2	1.9
	Medications	3	2.9	2	1.9
	Others	5	4.8	91	86.7
Dental History	Regular visit	81	77.1	80	76.2
	First visit	24	22.9	25	23.8

**Table 2:** The frequency among the case and control in relation to missing teeth status, number of teeth missing, and TMJ status.

		Case		Control	
		Frequency	Percent	Frequency	Percent
Teeth Missing	Unilateral	43	41.0	22	21.0
	Bilateral	37	35.2	26	24.8
	None	25	23.8	57	54.3
Number of Missing Teeth	2–3 teeth	44	41.9	21	20.0
	>3 teeth	36	34.3	27	25.7
	None	25	23.8	57	54.3
TMJ Affected	Right	31	29.5	0	0
	Left	29	27.6	0	0
	Both	45	42.9	0	0
	None	0	0	105	100.0

**Table 3:** The frequency of different pathologies among the case and the control group.

		Frequency	Percent
Case	Resorption	21	20.0
	Flattening	32	30.5
	Osteophytes	7	6.7
	Hyperplasia	14	13.3
	Cyst	1	1.0
	Normal	20	19.0
	Bifid	10	9.5
	Total	105	100.0
Control	Normal	105	100.0

**Table 4:** The frequency of bruxism among the case and the control group.

		Frequency	Percent
Case	Yes	18	17.1
	No	87	82.9
	Total	105	100.0
Control	Yes	1	1.0
	No	104	99.0
	Total	105	100.0

**Table 5:** Percentage of status of loss of teeth and number of teeth missing in the case and control group.

	Teeth Missing					*p* Value	Number of Teeth Missing			*p* Value
Groups			Unilateral	Bilateral	None	<0.001	2–3	>3	None	<0.001
	Case	Count	43	37	25		44	36	25	
		%within groups	41.0%	35.2%	23.8%		41.9	34.2	23.8	
		%within teeth missing	66.2%	58.7%	30.5%		67.7	57.1	30.5	
		% total	20.5%	17.6%	11.9%		21.0	17.1	11.9	
	Control	Count	22	26	57		21	27	57	
		%within groups	21.0	24.8	54.3		20.0	25.7	54.3	
		%within teeth missing	33.8	41.3	69.5		32.3	42.9	69.5	
		% total	10.5	12.4	27.1		10.0	12.9	27.1	

**Table 6:** Temporomandibular disorders among the case and control group.

			Right	Left	Both	None	*p* Value
groups	Case	Count	31	29	45	0	<0.001
		% within groups	29.5%	27.6%	42.9%	0.0%	
		% within TMJ Affected	100.0%	100.0%	100.0%	0.0%	
		% of Total	14.8%	13.8%	21.4%	0.0%	
	Control	Count	0	0	0	105	
		% within groups	0.0%	0.0%	0.0%	100.0%	
		% within TMJ Affected	0.0%	0.0%	0.0%	100.0%	
		% of Total	0.0%	0.0%	0.0%	50.0%	

**Table 7:** Percentage of pathology involved among the case and control group.

			Resorption	Flattening	Osteophytes	Hyperplasia	Cyst	Normal	Bifid	*p* Value
groups	Case	Count	21	32	7	14	1	20	10	<0.001
		% within groups	20.0%	30.5%	6.7%	13.3%	1.0%	19.0%	9.5%	
		% of Total	10.0%	15.2%	3.3%	6.7%	0.5%	9.5%	4.8%	
	Control	Count	0	0	0	0	0	105	0	
		% within groups	0.0%	0.0%	0.0%	0.0%	0.0%	100.0%	0.0%	
		% of Total	0.0%	0.0%	0.0%	0.0%	0.0%	50.0%	0.0%	

**Table 8:** Percentage of subjects affected with bruxism.

			Yes	No	Total	*p*-Value
Groups	Case	Count	18	87	105	<0.001
		% within groups	17.1%	82.9%	100.0%	
		% within Bruxism	94.7%	45.5%	50.0%	
		% of Total	8.6%	41.4%	50.0%	
	Control	Count	1	104	105	
		% within groups	1.0%	99.0%	100.0%	
		% within Bruxism	5.3%	54.5%	50.0%	
		% of Total	0.5%	49.5%	50.0%	

**Table 9:** Nonparametric Correlations show the correlation between different variables.

			Teeth Missing	Number of Missing Teeth	TMJ Affected	Pathology	Bruxism
Spearman’s rho	Groups	Correlation Coefficient	0.306 **	0.317 **	0.934 **	0.605 **	0.282 **
		Sig. (2-tailed)	<0.001	<0.001	<0.001	<0.001	<0.001
		N	210	210	210	210	210
	Age	Correlation Coefficient	−0.077	−0.061	−0.045	0.011	−0.142 *
		Sig. (2-tailed)	0.269	0.376	0.521	0.870	0.040
		N	210	210	210	210	210
	Gender	Correlation Coefficient	−0.051	−0.072	0.019	−0.027	0.005
		Sig. (2-tailed)	0.464	0.296	0.788	0.693	0.945
		N	210	210	210	210	210
	Medical History	Correlation Coefficient	−0.048	−0.074	−0.145 *	−0.004	−0.148 *
		Sig. (2-tailed)	0.488	0.285	0.036	0.953	0.032
		N	210	210	210	210	210
	Dental History	Correlation Coefficient	0.124	0.086	0.003	−0.035	−0.061
		Sig. (2-tailed)	0.073	0.214	0.967	0.619	0.375
		N	210	210	210	210	210
	Teeth Missing	Correlation Coefficient	1.000	0.916 **	0.281 **	0.157 *	0.067
		Sig. (2-tailed)	0.	<0.001	<0.001	0.023	0.334
		N	210	210	210	210	210
	Number of missing teeth	Correlation Coefficient	0.916 **	1.000	0.309 **	0.133	0.011
		Sig. (2-tailed)	<0.001	0.	<0.001	0.055	0.873
		N	210	210	210	210	210
	TMJ Affected	Correlation Coefficient	0.281 **	0.309 **	1.000	0.557 **	0.267 **
		Sig. (2-tailed)	<0.001	<0.001	0.	<0.001	<0.001
		N	210	210	210	210	210
	Pathology	Correlation Coefficient	0.157 *	0.133	0.557 **	1.000	0.310 **
		Sig. (2-tailed)	0.023	0.055	<0.001	0.	<0.001
		N	210	210	210	210	210
	Bruxism	Correlation Coefficient	0.067	0.011	0.267 **	0.310 **	1.000
		Sig. (2-tailed)	0.334	0.873	<0.001	<0.001	0.
		N	210	210	210	210	210

*. Correlation is significant at the 0.05 level (2-tailed). **. Correlation is significant at the 0.01 level (2-tailed).

**Table 10:** ODD ratio of bruxism among the case and control group.

Bruxism and Risk Estimate			
	Value	95% Confidence Interval	
		Lower	Upper
Odds ratio for groups (Case/Control)	21.517	2.816	164.443
For cohort Bruxism = Yes	18.000	2.447	132.395
For cohort Bruxism = No	0.837	0.765	0.914
N of Valid Cases	210		

**Table 11:** Significance and ODD ratio among different variables.

Category	Specification	Parameter	Significance	Exp(B)
TMJ Affected	Right	Intercept	0.956	
		Gender	0.151	0.474
		groups		2.159
	Left	Intercept	0.956	
		Gender	0.677	1.226
		groups		2.062
	Both	Intercept	0.956	
		Gender		1.433
		groups		2.051
Teeth Missing	Unilateral	Intercept	0.274	
		Gender	0.514	1.263
		groups	<0.001	0.223
	Bilateral	Intercept	0.395	
		Gender	0.181	1.635
		groups	0.001	0.300
Pathology	Flattening	Intercept	0.999	
		Gender	0.700	0.744
		groups	1.000	1.019
	Hyperplasia	Intercept	0.996	
		Gender	0.914	1.098
		groups	1.000	1.299

## Data Availability

All the data used in the study can be seen in the archieves of Ajman University Oral Radiology services.
